# Are an Aging Gut and a Decrease in Butyrate Production the Reasons for Atherosclerosis?

**DOI:** 10.3390/ijms26178276

**Published:** 2025-08-26

**Authors:** Leon M. T. Dicks

**Affiliations:** Department of Microbiology, Stellenbosch University, Stellenbosch 7600, South Africa; lmtd@sun.ac.za

**Keywords:** atherosclerosis, aging, gut microbiota

## Abstract

Atherosclerosis (AS) is directly linked to the aging and damage of endothelial cells (ECs). As ECs and vascular smooth muscle cells (VSMCs) age, more autocrine and paracrine signals are released, extending a vicious cycle of tissue aging and physiological dysfunction. The recruitment of immune cells to inflamed arteries, including coronary arteries, and an increase in the uptake of oxidised low-density lipoprotein (ox-LDL) by macrophages (foam cells) onto the tunica intima (intima) of coronary arteries restrict blood flow. The inability of aging and damaged ECs to accommodate vast changes in signalling molecules, many produced by gut microbiota, leads to a range of anatomical and physiological arterial anomalies. These include degradation of cardiovascular membranes, fibrosis, calcification, plaque formation, and an increasingly dysfunctional immune system. Changes in the gut microbiome of the elderly have a direct effect on the immune response, as the signalling molecules produced by gut microbiota target specific receptors on inflamed arteries. This review summarizes the anatomical and physiological changes associated with the aging of coronary arteries and emphasizes the conditions leading to AS. The importance of butyrate-producing gut microbiota in preventing AS, especially in the elderly, is discussed.

## 1. Introduction

Aging may be defined as a progressive change in biological processes, driven by genomic instability and a decline in cellular communication. Modulation in the sensing of nutrients by a changing gut microbiome, variations in inflammatory reactions, the uncontrolled migration of macrophages, loss of enzymatic activities, and a decline in mitochondrial functions are some of the anomalies associated with aging [1]. These changes are key drivers of illnesses such as atherosclerosis (AS), associated with, but not limited to, the elderly [2], metabolic disease, neurodegenerative disorders, and cancer [3,4,5,6]. The gradual decline in physiological activities of intestinal epithelial cells (IECs) and the disappearance of signal-specific membrane receptors lead to an imbalance in the expression of pro- and anti-inflammatory cytokines, and drastic changes in the gut microbiome [3,7].

**Figure 1:**
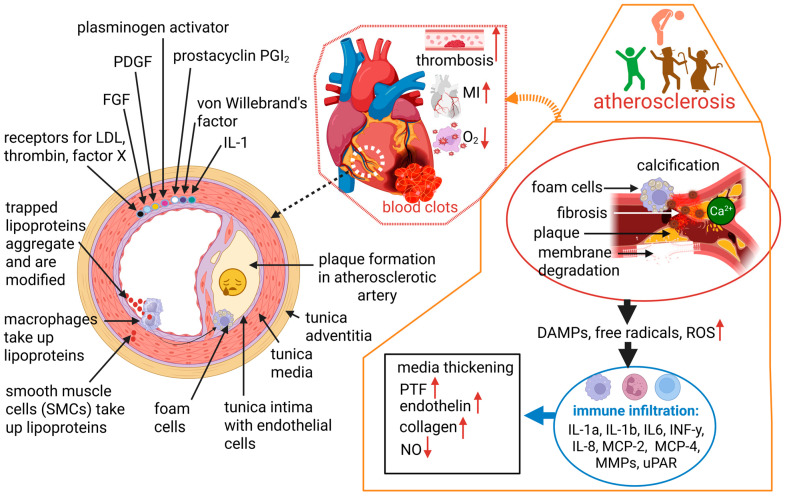
Persistent or chronic inflammation, typical in the elderly, leads to atherosclerosis (AS), characterized by the infiltration of immune cells into vascular smooth muscle cells (VSMCs, also referred to as smooth muscle cells, SMCs), and an increase in collagen, endothelin, and protein tissue factor (PTF), which leads to the thickening of the media. These changes in the arterial wall initiate the release of damage-associated molecular patterns (DAMPs), free radicals, and reactive oxygen species (ROS), which enhance inflammation. This increases the risk of blood clotting, thrombosis, a decrease in oxygen (O_2_) supply to the myocardium, and myocardial infarction (MI). The uptake of oxidised low-density lipoprotein (ox-LDL) by macrophages results in the formation of foam cells that adhere to the intima and contribute to plaque formation and restriction of blood flow. The surface of the intima of healthy coronary arteries is covered with receptors for LDL and thrombin, prostacyclin PGI_2_ (an antithrombotic agent), von Willebrand’s factor (a prothrombotic agent), interleukin-1 (IL-1), and plasminogen activator (a fibrinolytic agent). Damaged blood vessels are repaired by platelet-derived growth factor (PDGF) and fibroblast growth factor (FGF) released from platelets. NO = nitric oxide, IL = interleukin, INF-y = interferon y, MCP = monocyte chemoattractant protein, MMPs = matrix metalloproteinases, uPAR = urokinase plasminogen activator receptor. Red arrows showing upwards denotes an increase and downward arrows a decrease. The schematic representation was constructed using Biorender (Biorender.com), accessed on 14 July 2025.

The inability of endothelial cells (ECs) in the intima of coronary arteries to accommodate vast changes in signalling molecules, many produced by gut microbiota, leads to a range of anatomical and physiological anomalies, e.g., calcification, immune infiltration, degradation of cardiovascular membranes, deposition of lipid-rich macrophages (foam cells), plaque formation, and fibrosis, as summarized in Figure 1 [8]. Apart from plaque formation, blood flow is disrupted, or arteries are completely clogged, which may lead to thrombosis and myocardial infarction (MI) (Figure 1). Prolonged inflammation leads to irreversible tissue failure, which is often difficult to correct with conventional therapies [9].

Arterial inflammation is characterized by the translocation of nuclear and mitochondrial DNA (mtDNA) into the cytosol [10]. This stimulates pro-inflammatory DNA sensors, exaggerated by ineffective autophagy (the degradation and recycling of cellular components), inadequate gene regulations, the incorrect folding of proteins, and malfunctioning of enzymes [3]. Damaged ECs stimulate the release of inflammatory cytokines, e.g., interleukin (IL)-1a, IL-1b, IL6, and interferon y (INF-y), chemokines, e.g., IL-8, monocyte chemoattractant protein 2 (MCP-2), and MCP-4, and matrix metalloproteinases (MMPs); and growth regulators, such as the urokinase plasminogen activator receptor (uPAR). These are listed in Figure 1 [11]. These relapses of membrane structure stimulate the release of damage-associated molecular patterns (DAMPs), free radicals, and reactive oxygen species (ROS), which enhance inflammation (Figure 1) [12]. Damaged blood vessels are repaired by platelet-derived growth factor (PDGF) and fibroblast growth factor (FGF) released from platelets (Figure 1) [13]. Leukocytes, such as IL-1, are recruited (Figure 1) and trigger the expression of protein tissue factor (PTF), which acts as a procoagulant [14].

In a previous review [8], the production of butyrate by gut microbiota, the uptake of butyrate by colonic endothelial cells, transport across arterial and myocardial endothelia, and interactions with G-protein-coupled receptors (GPCRs) were discussed. This review summarizes the anatomical, physiological, and molecular changes associated with the aging of coronary arteries. Cellular abnormalities and the role that gut microbiota play in the development of AS are discussed. The review is not a detailed discussion of changes in the gut microbiome at different ages. The focus is on microbial signals generated by gut microbiota, especially those produced by an “aging gut microbiome”. The importance of butyrate-producing gut microbiota in preventing AS is discussed.

## 2. Changes in the Vessel Wall

One of the hallmarks of AS and other forms of cardiovascular diseases (CVDs) is the dysfunction of ECs [15]. Persistent inflammation triggers genomic instability and somatic mutations, which may lead to an increase in the production of a specific clone of blood cells, referred to as clonal haematopoiesis (CH). This initiates AS and may cause certain blood cancers, e.g., myeloid leukaemia, myelodysplastic syndrome, and myeloproliferative neoplasms [16]. The destruction of coronary arteries by persistent inflammation leads to a decline in the density of capillaries and arterioles, a response typical of arteriosclerotic arteries and a precursor of atherothrombosis [17]. These changes coincide with an increase in coronary collagen and endothelin, loss of membrane elasticity, a decrease in nitric oxide (NO) production, and thickening of the medial layer (media) in coronary arteries (Figure 1) [17].

**Figure 2:**
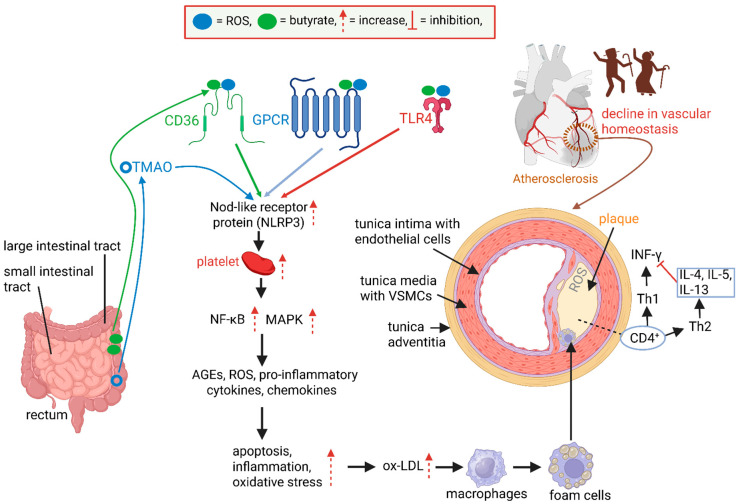
Butyrate and reactive oxygen species (ROS), produced by microbiota in the large intestinal tract, interact with Toll-like receptor 4 (TLR4), platelet glycoprotein 4 (CD36), and G-protein-coupled receptors (GPCRs). Trimethylamine-N-oxide (TMAO), produced by gut microbiota, activates Nod-like receptor protein 3 (NLRP3) and triggers platelet formation. This results in the stimulation of the nuclear factor kappa B (NF-κB) and mitogen-activated protein kinase (MAPK) pathways, which increases the expression of pro-inflammatory cytokines, chemokines, advanced glycation end products (AGEs), and reactive oxygen species (ROS). These increases lead to an increase in apoptosis, inflammation, oxidative stress, and oxidised low-density lipoprotein (ox-LDL). The latter is taken up by macrophages, which convert to foam cells that adhere to the tunica intima. The result is plaque buildup and restriction of blood flow. Plaques upregulate CD4^+^, which differentiates into helper T (Th) cells, Th1 and Th2. Th1 cells express interferon-γ (INF-γ) to promote atherosclerosis (AS), whilst Th2 cells produce IL-4, IL-5, and IL-13, which neutralize INF-γ. The schematic representation was constructed using Biorender (Biorender.com), accessed on 14 July 2025.

The surface of the intima of healthy coronary arteries is covered with receptors for LDL and thrombin, prostacyclin PGI_2_ (an antithrombotic agent), von Willebrand’s factor (a prothrombotic agent), interleukin-1 (IL-1), and plasminogen activator (a fibrinolytic agent) (Figure 1) [18]. Damaged blood vessels are repaired by platelet-derived growth factor (PDGF) and fibroblast growth factor (FGF) released from platelets (Figure 1) [19]. Leukocytes, such as IL-1, are recruited (Figure 1) and trigger the expression of protein tissue factor (PTF), which acts as a procoagulant [14]. A combination of these factors maintains blood flow in damaged arteries, prevents the formation of blood clots, and protects the intima from plaque buildup. Short-chain fatty acids (SCFAs), e.g., butyrate, and reactive oxygen species (ROS) produced by gut bacteria interact with signaling pathways and cell membrane receptors, e.g., Toll-like receptor 4 (TLR4), platelet glycoprotein 4 (CD36), and G protein-coupled receptors (GPCRs), as shown in Figure 2 [18]. Trimethylamine-N-oxide (TMAO), produced by gut microbiota, activates Nod-like receptor protein 3 (NLRP3) and triggers platelet formation (Figure 2) [18]. Gut bacteria such as *Escherichia* and *Klebsiella* are known to increase TMAO levels [19].

The decline of vascular homeostasis destabilizes membrane permeability, increases Nod-like receptor protein 3 (NLRP3), stimulates the nuclear factor kappa B (NF-κB) pathway, and induces apoptosis (Figure 2). An increase in NLRP3 activates NF-κB and mitogen-activated protein kinase (MAPK) pathways, which in turn lead to an increase in the expression of cytokines and chemokines (Figure 2) [18,20]. As a result of these changes, the levels of AGEs (advanced glycation end products) and ROS increase (Figure 2) [21,22]. An increase in oxidative stress leads to the production of ox-LDL, which is taken up by macrophages and transformed into lipid-rich foam cells (Figure 2). The latter accumulate on the intima and participate in plaque formation (Figure 2) [18]. T- and B-lymphocytes are also involved in the development of AS [23]. The main T cells in atherosclerotic plaques are CD4^+^ (Figure 2) [24]. Upon activation, CD4^+^ differentiate into helper T (Th) cells. Th1 cells express interferon-γ (INF-γ) to promote AS, whilst Th2 cells produce IL-4, IL-5, and IL-13, which neutralize INF-γ (Figure 2) [25]. Vascular smooth muscle cells (VSMCs) are maintained by Notch 3, which regulates gene transcriptions, intracellular communication, neural development, and the binding of epidermal growth factor (EGF) to EGF receptors (EGFRs) [26]. Arteries prone to AS are penetrated by foam cells, accumulate ROS, and are more prone to fibrosis (Figure 2) [27].

**Figure 3:**
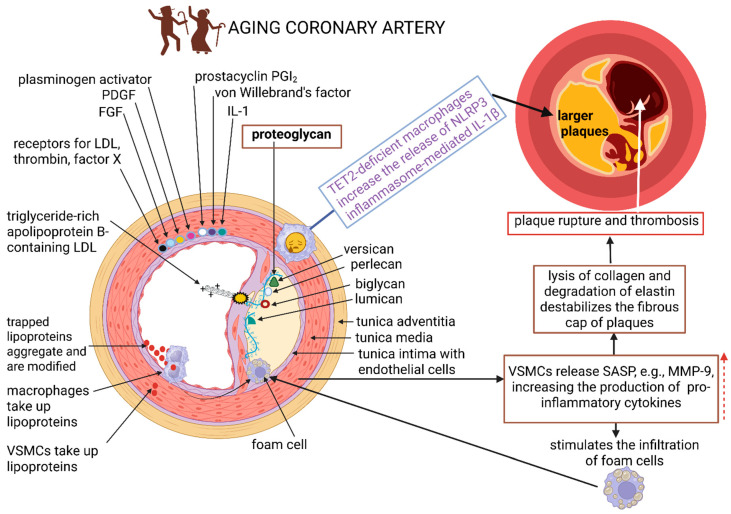
The tunica intima (intima) of aging endothelial membranes with AS is covered by proteoglycans. The negatively charged side chains, biglycan, versican, perlecan, and lumican, are linked to positively charged apolipoprotein B attached to oxidised low-density lipoprotein (ox-LDL). Senescence-associated secretory phenotype (SASP) factors, e.g., matrix metalloproteinase 9 (MMP-9) released from vascular smooth muscle cells (VSMCs), upregulate the inflammasome and stimulate the infiltration of foam cells into arteries. With the increase in MMP production, collagen is lysed and elastin degraded, destabilizing the fibrous cap of plaques. The rupture of plaques increases the risk of thrombosis. TET methylcytosine dioxygenase 2 (TET2)-deficient macrophages, prevalent in ageing blood cells, increase the release of Nod-like receptor protein 3 (NLRP3) inflammasome-mediated interleukin-1β (IL-1β), resulting in the formation of larger plaques. The red dashed arrow denotes an increase. The schematic representation was constructed using Biorender (Biorender.com), accessed on 14 July 2025.

**Figure 4:**
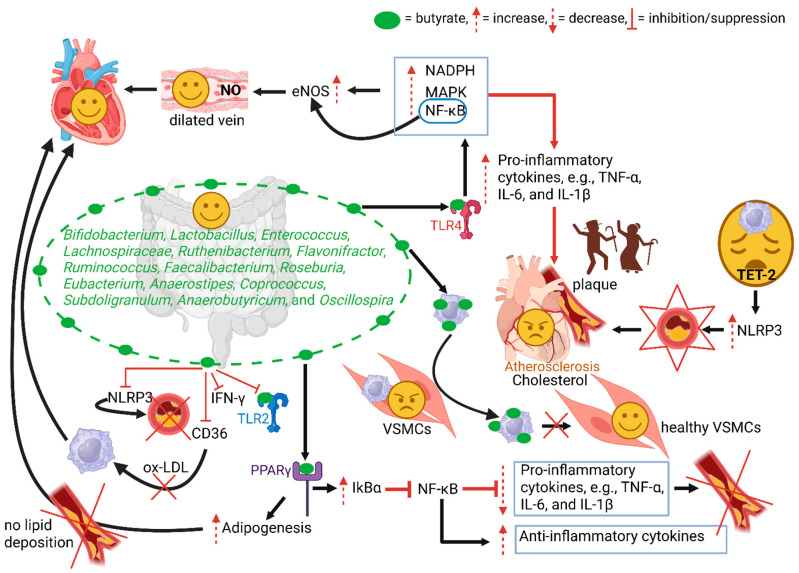
Butyrate prevents the infiltration of macrophages into VSMCs and suppresses the production of pro-inflammatory cytokines, thereby preventing plaque formation. Binding of butyrate to peroxisome proliferator-activated receptor γ (PPARγ) increases the activity of IkBα (nuclear factor kappa-B, NF-κB, inhibitor). This suppresses the production of pro-inflammatory cytokines that cause atherosclerosis (AS). Binding to PPARγ also stimulates adipogenesis, preventing plaque formation and AS. Binding butyrate to Toll-like receptor 4 (TLR4) increases the production of pro-inflammatory cytokines and induces plaque formation. Larger plaques are formed when TET methylcytosine dioxygenase 2 (TET-2)-deficient macrophages stimulate the release of Nod-like receptor protein 3 (NLRP3). Butyrate suppresses inflammation by inhibiting interferon γ (IFN-γ), TLR2, and platelet glycoprotein CD36 signalling. By suppressing CD36, the uptake of oxidised low-density lipoprotein (ox-LDL) by macrophages is prevented, and plaque formation is repressed. NADPH = nicotinamide adenine dinucleotide phosphate, MAPK = mitogen-activated protein kinase, eNOS = endothelial nitric oxide synthase, NO = nitric oxide, VSMCs = vascular smooth muscle cells. The schematic representation was constructed using Biorender (Biorender.com), accessed on 14 July 2025.

In ageing endothelial membranes with AS, the intima is covered by proteoglycans (Figure 3). Their negatively charged side chains (biglycan, versican, perlecan, and lumican) are linked to positively charged lipoproteins, specifically apolipoprotein B, which attaches to ox-LDL (Figure 3) [28]. Aging VSMCs release senescence-associated secretory phenotype (SASP) factors, e.g., matrix metalloproteinase 9 (MMP-9), and upregulate the inflammasome, preparing adjacent healthy endothelial cells and smooth muscle cells to produce pro-inflammatory cytokines (Figure 3) [21,29]. This stimulates the infiltration of foam cells (Figure 3) [30]. With the increase in MMP production, collagen is lysed and elastin degraded, destabilizing the fibrous cap of plaques, risking rupture, the release of plaque contents, and thrombosis (Figure 3) [21,30]. An aging immune system with impaired function may fail to eliminate dead cells and support the release of SASP [7]. Senescent immune cells infiltrate vessel walls, leading to an increase in the production of pro-inflammatory mediators and chemokines [7]. TET methylcytosine dioxygenase 2 (TET2)-deficient macrophages, prevalent in ageing blood cells, increase the release of NLRP3 inflammasome-mediated interleukin-1β (IL-1β) (Figure 3 and Figure 4) [21]. This results in the formation of larger aortic plaques (Figure 3 and Figure 4) [31].

Ox-LDL accelerates the degradation of macrophages, promotes the release of pro-inflammatory cytokines, and prevents macrophage migration [32]. A degradation of CD4^+^ and CD8^+^ T cells, correlated with a decline in IFN-γ and TNF-α and an increase in plaque infiltration, leads to the expression of perforin and granzymes, which damage surrounding ECs and VSMCs [24]. Further research is required to determine whether the accumulation of senescent T cells in peripheral blood cells and atherosclerotic plaques is the cause or consequence of atherogenesis.

## 3. Changes in the Gut Microbiome

The adult human gut hosts approximately 4 trillion microorganisms, equivalent to the estimated 3.0 × 10^13^ human cells in a 70 kg body [33]. The human gut hosts six prominent phyla, i.e., Bacillota (previously Firmicutes), Bacteroidota (previously Bacteroidetes), Pseudomonadota (previously Proteobacteria), Fusobacteriota (previously Fusobacteria), Verrucomicrobiota (previously Verrucomicrobia), Cyanobacteriota (previously Cyanobacteria), and Actinomycetota (previously Actinobacteria) [34]. Bacillota and Bacteroidota are predominant [34]. Fungi are less diverse, with *Candida*, *Saccharomyces*, *Malassezia*, and *Cladosporium* being the best represented. [34]. Less is known about the virome of the human gut, except for pathogenic viruses, such as *Enterovirus* from the family *Picornaviridae* [35], and *Rotavirus* [36] and *Norovirus* [37], from the families *Reoviridae* and *Caliciviridae*, respectively. Other members of the human gut virome are from the families *Picobirnaviridae*, *Astroviridae*, *Adenoviridae*, *Anelloviridae*, *Cycloviridae*, and *Parvoviridae* [38]. The diversity of bacteria within the human gut was reviewed by Rosenberg [39].

At birth, the infant is exposed to the mother’s microbiome. The exchange of microbiota is highly dependent on the delivery of the newborn [40]. The gastrointestinal tract (GIT) of infants born by Caesarean (C)-section is invaded by *Enterobacter hormaechei*, *Enterobacter cancerogenus*, *Haemophilus parainfluenzae*, *Haemophilus aegyptius*, *Haemophilus influenza*, *Haemophilus haemolyticus*, *Staphylococcus saprophyticus*, *Staphylococcus lugdunensis*, *Staphylococcus aureus*, *Streptococcus australis*, *Veillonella dispar*, *Veillonella parvula*, and a few *Bacteroides* spp. [41]. A comparative study conducted on same-age infants vaginally delivered showed less bacterial diversity, with dominance by *Bacteroides*, *Bifidobacterium*, *Parabacteroides*, *Escherichia*, and *Shigella* [42]. The core group of gut microbiota develops within the first 5 years [43]. Later in life, closer to puberty, the gut microbiome adapts to dietary preferences, external factors, and treatments of illnesses [44].

A diet rich in fat and carbohydrates (Western-style diet, WD) supports the growth of Bacillota (e.g., *Streptococcus* and *Oscillibacter*), Pseudomonadota, and Fusobacteriota, but suppresses the growth of *Lactobacillus* [45]. In general, individuals following an imbalanced WD are more obese and harbour more Bacillota and Bacteroidota compared to individuals on a Mediterranean diet (MD) [43,46]. Reports are not always consistent. According to Severino et al. [45], a WD is less supportive of Bacteroidota (e.g., *Bacteroides*, *Parabacteroides Prevotella*, *Barnesiella*, and *Alistipes*) and Actinomycetota (e.g., *Bifidobacterium*, *Streptomyces*, and *Actinoplanes*). According to Desai et al. [47], a WD promotes the growth of mucus-degrading *Akkermansia muciniphila* and *Bacteroides caccae*. The destruction of the mucus layer may render IECs vulnerable to pathogens such as *Helicobacter pylori*. Since *H. pylori* regulates the NLRP3 inflammasome [48], infection may initiate AS. Other bacteria associated with AS are *Escherichia coli* [49] and *Acidaminococcus* [50]. Diets with a high fiber content support the growth of *Bifidobacterium*, *Lactobacillus*, *Enterococcus*, *Lachnospiraceae*, *Ruthenibacterium*, *Flavonifractor*, *Ruminococcus* [51], *Faecalibacterium*, *Roseburia*, *Eubacterium*, *Anaerostipes*, *Coprococcus*, *Subdoligranulum*, *Anaerobutyricum*, and *Oscillospira* [52].

A comprehensive study conducted by Odamaki et al. [53], based on the sequencing of the *16S rRNA* genes (V3-V4 regions) of more than 1.8 million bacteria isolated from individuals of various age groups (newborn infants to individuals 104 years old), has shown an increase in Bacteriodota and Pseudomonadota, and a decline in Bacillota in individuals older than 70 years. The dominant Bacillota were *Eubacterium*, *Clostridium*, *Megamonas*, *Peptoniphilus*, *Lachnospiraceae*, *Blautia*, *Coprococcus*, *Roseburia*, and *Faecalibacterium* [54]. According to Salazar et al. [55], centenarians harbour less Bacillota and more Bacteroidota and *Proteus* spp. This may be due to an increase in inflammation as the immune system becomes compromised with changes in diet [56]. The gut microbiota of the elderly is more adapted to proteolytic activities than glycolysis [56]. This promotes the growth of pathogenic bacteria and aggravates inflammatory responses, e.g., an increase in ROS. An increase in ROS inactivates strict anaerobic bacteria (i.e., Bacillota) and promotes facultative aerobes [57]. With an increase in the expression of inflammatory cytokines, the expression of tight junction proteins is reduced, and the gut wall becomes more permeable. This further increases inflammation [58].

A reduction in *Bifidobacterium* in the elderly [53] is influenced by diet [59]. Bifidobacteria downregulate pro-inflammatory responses in the gut epithelium [60] and produce acetate, which stimulates the growth of butyrate-producing bacteria [61]. It is interesting to note that individuals diagnosed with AS have less butyrate-producing *Bifidobacterium*, *Bacteroides*, *Alistipes*, *Parabacteroides*, and *Prevotella* [62]. Instead, they have significantly more Bacillota, especially members of the genera *Megamonas*, *Streptococcus*, and *Veillonella* [62]. *Megamonas* is associated with obesity, several metabolic diseases, and inflammation [63]. *Veillonella* is associated with insulin resistance [64]. *Streptococcus* has been isolated from atherosclerotic plaques [65]. Bifidobacteria strengthen gut barrier integrity and reduce systemic inflammation [66,67,68]. A decline in bifidobactera may explain why these individuals are more prone to developing AS.

## 4. Molecular Changes Driven by Gut Microbiota

With age and deterioration of renal function, TMAO levels increase [69], the population of SCFA-producing bacteria decreases [70], and the composition of bile acid changes [71]. Trimethylamine N-oxide (TMAO), produced by gut microbiota, activates nitric oxide dismutase leucine-rich repeats and pyrin 3 (NLRP3) inflammatory bodies, increases the release of intracellular calcium ions [72], accelerates the formation of aortic lesions by activating Farnesoid X receptors (FXRs) [73], and activates the protein kinase C/NF-kB (canonical NF-kB)/vascular cell adhesion molecule-1 pathway. At the same time, TMAO inhibits the ROS-thioredoxin interactive protein axis [74]. All these reactions promote AS [75]. TMAO also upregulates CD36 expression and the ATP-binding cassette transporter A1 in macrophages, resulting in the accumulation of cholesterol [76], and alters the electrophysiology in arteries [77].

Mucin production generally decreases with age as goblet cells become inactive [78]. Drastic changes in the expression of mucin may lead to an imbalance in microbiota, overgrowth of pathogens, changes in anti-inflammatory regulatory networks, and, ultimately, intestinal disease [79]. Sialic acid Neu5AC, produced by IECs, regulates the texture of mucus and is thus indirectly responsible for the adhesion of microorganisms [80]. Commensal gut bacteria stimulate mucin glycan production and the formation of new goblet cells. Apart from keeping the GIT in a homeostatic state, mucus also prevents the apoptosis of IECs and helps to maintain the integrity of the gut wall [80]. Other changes that occur with an aging gut are the loss of interstitial cells of Cajal [81], a reduction in cholinergic motor nerve functions [82], loss of enteric glial cells [83], an increase in senescence-like activity within myenteric nerve cell bodies [84], and an increase in total collagen content within muscles and the submucosal plexus [85].

Toll-like receptors and C-type lectin receptors on cell surfaces and the cytosolic nucleotide-binding oligomerisation domain, also known as NLRs, play a role in compartmentalisation and segregation of the commensal microbiota [86]. NLRs sense microbial infection through the recognition of MAMPs, which leads to activation of the innate immune response and the upregulation of key pro-inflammatory pathways, including interleukin (IL)-1β production and translocation of nuclear factor-κB (NF-κB) to the nucleus [87]. Activation of NF-κB leads to an increase in the expression of genes that regulate inflammatory and immune responses. Typical examples are the production of IL-8 and the pro-inflammatory mediator tumour necrosis factor α (TNF-α), which in turn results in the recruitment and activation of neutrophils [87]. TNF-α is synthesised by Paneth cells, located adjacent to the stem cell zone in the base of the crypts of Lieberkühn in the small intestine. Paneth cells also secrete microbicidal peptides (predominantly cryptdins) when in contact with Gram-negative or Gram-positive bacteria, lipopolysaccharide (LPS), lipoteichoic acid (LTA), lipid A, and muramyl dipeptide (MDP), a peptidoglycan fragment [88]. MDP activates NOD2, which in turn induces Paneth cells to produce defensins, i.e., small cysteine-rich cationic antimicrobial proteins [89]. Activation of NOD1 receptors by peptidoglycan stimulates lymphoid follicles and activates neutrophils [89,90]. The importance of NOD in the regulation of the gut microbiome is evident from studies that showed a deficiency in NOD2 correlated with increased numbers of Bacteroidota and Bacillota [90].

Antigens that cross the epithelial barrier interact with immune cells in the lymphoid tissue, induce an immune response, and are then transported from the intestinal lumen across the epithelial cell layer using enterocytes, M-cells, and DC pathways [91]. Antigen-presenting DCs are in direct contact with commensal microbiota and respond by the expression of TLRs and NLRs, activating naïve T-cells into effector T-cells (TH1, TH2, and TH17) or Treg [92]. An increase in lipopolysaccharide (LPS)-producing bacteria in the elderly activates TLRs and increases the degradation of mucous layers. Failure to maintain the defence against pathogens leads to changes in the permeability of IECs, which may lead to the development of bacteraemia and inflammation of the gut wall [93]. Paneth cells are induced by commensal strains, such as *Bacteroides thetaiotaomicron*, to produce angiogenin-4 (Ang4), which has antibacterial activity [94]. A decline in Bacteriodota, as observed in individuals with AS, may compromise the production of Ang4.

Production of IL-18 is crucial to repair epithelial cells and maintain the epithelial cell barrier [95]. The inflammasome is activated by SCFA-producing bacteria and activates CASPASE-1, a protease responsible for processing pro-IL-1β and pro-IL-18 [80]. Immune and epithelial cells express NLRP3 and NLRP6 inflammasomes, respectively. The NLRP6 epithelial inflammasome prevents microbial dysbiosis and development of inflammatory diseases such as ulcerative colitis by the production of IL-18. Pro-inflammatory cytokines induce TH1 and TH17 immune responses and activate neutrophils and macrophages to prevent infection. The activation of CASPASE-1 may also lead to pyroptosis, which is cell death with combined characteristics of necrosis and apoptosis. However, stimuli causing the activation of inflammasomes, and subsequently CASPASE-1, do not necessarily lead to CASPASE-1-associated epithelial cell death [96].

Early stages of blood clotting are initiated by the interaction between plasma kallikrein and factor XII, resulting in the release of the nanopeptide bradykinin. This increases the permeability of arterial walls, leading to leakage and inflammation, as reviewed by Dicks [18]. Intestinal health and lipid oxidation play critical roles in CVD [18]. Plasminogen activator inhibitor (PAI)-1, produced by endothelial cells (ECs), VSMCs, adipocytes, and platelets, decreases fibrinolysis and promotes clot formation. Urokinase plasminogen activator (uPA), a serine protease, converts inactive plasminogen into active plasmin. The protein epidermal growth factor receptor (EGFR) is involved in cell signaling pathways, cell division, and survival. Paraoxonases (PONs), especially PON1, protect LDL and outer cell membranes against oxidative modifications. PON2 lowers ROS, represses apoptosis, and protects mitochondria against oxidative stress [18]. PON3 interacts with apolipoprotein A-1(apoA1)-HDL, in a similar process to that described for PON1, and is strongly associated with microbial diversity and their metabolic pathways, especially the biosynthesis of antioxidant vitamin B1 [18].

Epithelial cellular adhesion molecule (Ep-CAM) and trefoil factor 3 (TFF3), a small peptide, secreted by intestinal goblet cells, keep intestinal epithelial cells (IECs) fused, prevent mucosal infections [97], and help repair the intestinal mucosa [98]. However, more recent research has linked TFF3 to cancer, colitis, gastric ulcers, diabetes mellitus, non-alcoholic fatty liver disease (NAFLD), and abnormalities of the CNS [98]. We do know that changes in Ep-CAM levels have an impact on the production of alpha-1,2-fucosyltransferase 2 (FUT2) and influence the composition of the gut microbiome [99].

A recent study [100] has shown that imidazole propionate (ImP), produced by gut microbiota, induced AS without altering lipid compositions. Supplementation of diets with ImP increased the development of AS in the aorta and aortic root without affecting circulating cholesterol or glucose concentrations. The binding of AS to the imidazoline-1 receptor (I1R, also known as nischarin), present in myeloid cells, induced phosphorylation of ribosomal protein S6 (pS6), a downstream target of the mammalian target of the Rapamycin (mTOR) pathway. mTOR, a protein kinase, is involved in regulating cellular metabolism and growth, and survival of macrophages [101,102]. The activation of mTORC1 in macrophages increases inflammation, apoptosis (programmed cell death), and impaired autophagy (cellular recycling), leading to the initiation of AS [103]. mTORC2, on the other hand, plays a protective role in AS by regulating cell survival and restraining the activity of MST1, a serine/threonine kinase and a key component of the Hippo signaling pathway, which contributes to cell death [103]. Furthermore, ImP activated systemic and local innate and adaptive immunity and inflammation [100]. Single-cell RNA-sequencing (scRNA-seq) of aortas from mice treated with ImP for eight weeks showed an increase in fibroblasts, endothelial cells, and immune cells [100]. The increase in immune cells was confirmed by flow cytometry and histological analysis of the aortic root [100]. In mice treated with ImP, clear signs of T cell infiltration in the intima–media layer and an increase in inflammatory macrophages were observed in plaque [100]. In vitro studies on mouse embryonic fibroblasts (MEFs) indicated that ImP activated embryonic fibroblasts by increasing the levels of monocyte chemoattractant protein-1 (MCP-1). The latter recruits monocytes associated with atherogenesis [104]. An increase in bone marrow-derived macrophages (BMDMs) and MEFs suggested that macrophages and fibroblasts are the main targets of ImP [102]. In another study [105], an increase in *Clostridium leptum* and pathogenic *Enterobactericeae* was noted in the gut microbiome of elderly people diagnosed with AS. The same study also reported an increase in pathogenic *Streptococcus* spp. in coronary plaques from the same group of individuals. Earlier studies [106,107] reported the presence of Pseudomonadota (e.g., *Enterobacter* spp.), *Cryseomonas* spp., *Veillonella* spp., *Staphylococcus* spp., *Propionibacterium* spp., and *Chlamydia* spp. in plaques from AS patients. Since these species are endemic to the human gastrointestinal tract [105], these findings suggest that the bacteria translocate to coronary arteries and accumulate in plaque. This may lead to the production of endotoxins in plaque [105]. This is troublesome, as Pseudomonadota and *Escherichia coli* levels are known to increase in the gastrointestinal tracts (GITs) of the elderly.

## 5. Immunosenescence in Aging and Atherosclerosis

Immunosenescence refers to the ageing of the immune system. This process is characterized by a decline in adaptive immunity and regulation of antigen-specific processes, specifically a reduction in B- and T cells, as well as changes in antibody production [23]. Aging of the immune system causes an imbalance in hematopoietic stem cells (HSCs), leading to myelopoiesis and a diminished lymphoid cell pool [108]. With aging, fewer naïve T and B cells are produced from bone marrow, and the production of lymphoid progenitor cells declines [109]. Although a reduction in the range of B-cell receptors (BCRs) was reported in the peripheral blood and lymph nodes of elderly individuals, an increase in BCR diversity was noted in spleens of the elderly [110,111,112]. These conflicting results may be due to variations in the ability of aged B cells to respond to IL-7, which they require for survival [113,114]. Adaptation to the SASP leads to the release of MMP-9 by ASMCs and an upregulation of the inflammasome [21,29]. Smooth muscle cells and healthy ECs increase the release of pro-inflammatory cytokines, which results in chronic low-level inflammation, referred to as “inflammaging” [115].

AS, normally associated with the elderly, is a lipid-driven chronic inflammatory process [114]. Damaged endothelia are infiltrated by lipids and immune cells, leading to plaque formation [8,18], a disruption in blood flow, thrombosis, and myocardial infarction (MI) [9]. CD4^+^ cells differentiate into helper T (Th) cells, with Th1 cells expressing interferon-γ (INF-γ) [25]. Th2 cells produce IL-4, IL-5, and IL-13, which neutralize INF-γ, as summarized in Figure 2 [25]. TET-2-deficient macrophages release NLRP3 inflammasome-mediated IL-1β, which increases the size of plaques [116]. Changes in the gut microbiome, which are inevitable with aging, change the expression of TLRs and NLRs by DCs in direct contact with gut microbiota. Naïve T-cells transgress to effector T-cells (TH1, TH2, and TH17) or Treg [92]. Regulatory T cells (Tregs) produce IL-10 and TGF-β, which inhibit pro-atherogenic effector T cells and stimulate efferocytosis by macrophages [117]. In ageing endothelial membranes, as with AS, negatively charged side chains (biglycan, versican, perlecan, and lumican) are linked to positively charged lipoproteins, specifically apolipoprotein B, which attaches to ox-LDL (Figure 3) [28]. Tregs may act autoreactively to apolipoprotein B and help suppress inflammation, thus preventing AS. With the progression of AS, the protective role of Tregs may decline, and they may produce pro-inflammatory cytokines, which stimulate immune responses against apolipoprotein and aggravate plaque formation [118]. AS is also associated with the accumulation of damaged DNA, as specifically noted in peripheral blood lymphocytes. This correlates with diabetes and hypercholesterolemia [119]. For further information on adaptive immunity, immunosenescence, and AS, the reader is referred to the comprehensive reviews by Snijckers and Foks [114] and Liu et al. [120].

## 6. The Link Between Butyrate and Atherosclerosis (AS)

An increase in Bacillota (associated with high caloric intake) and a decrease in Bacteroidota (associated with anti-inflammatory effects) may stimulate the production of proinflammatory cytokines and increase cholesterol levels, both of which are risk factors for AS [46,121]. Several studies have shown that butyrate-producing bacteria such as *Bifidobacterium*, *Lactobacillus*, *Enterococcus*, *Lachnospiraceae*, *Ruthenibacterium*, *Flavonifractor*, *Ruminococcus*, *Faecalibacterium*, *Roseburia*, *Eubacterium*, *Anaerostipes*, *Coprococcus*, *Subdoligranulum*, *Anaerobutyricum*, and *Oscillospira* activate the TLR4 pathway, increase nicotinamide adenine dinucleotide phosphate (NADPH), and activate NF-κB and MAPK pathways, as summarised in Figure 4 [52,54,122]. Bacteria associated with AS are listed in Table 1. Bacterial DNA may also trigger macrophages and activate the innate immune system via TLR2 and TLR4 [123,124]. The upregulation of these pathways leads to an increase in proinflammatory cytokines, and the formation of plaques (Figure 4) [125]. By increasing NF-κB, endothelial nitric oxide synthase (eNOS) is activated, and NO levels increase (Figure 4). NO relaxes VSMCs and may ease the restriction of arteries [126].

Butyrate prevents the infiltration of macrophages into VSMCs (Figure 4) [23,127] and decreases the production of pro-inflammatory cytokines, which suppresses plaque formation (Figure 4) [8,128]. Depending on the inflammatory stimuli, butyrate may inactivate NF-κB, suppress proinflammatory cytokines, and stimulate anti-inflammatory cytokines (Figure 4). Butyrate may regulate MAPK to suppress inflammation, muscle cell growth, apoptosis, and the uptake of ox-LDL by macrophages [8,129]. Binding of butyrate to peroxisome proliferator-activated receptor γ (PPARγ) increases the activity of IkBα, thereby inhibiting the production of NF-κB (Figure 4) [8]. This, along with the ligation of PPARγ with p65, degrades the NF κB/p65 complex [8], resulting in downregulation of the NF-κB pathway [8], the suppression of pro-inflammatory cytokines, and an increase in anti-inflammatory cytokines (Figure 4) [8,128]. Butyrate may also exert anti-inflammatory activities by inhibiting interferon γ (IFN-γ) signalling (Figure 4). This suppresses the activity of CD36 and decreases the uptake of ox-LDL by macrophages (Figure 4) [8,128,129]. Furthermore, butyrate suppresses the NOD-, LRR-, and pyrin domain-containing protein 3 (NLRP3) inflammasome, which alleviates inflammation and scar tissue formation (Figure 4) [8]. The opposite is true for TET-2-deficient macrophages. They increase the release of NLRP3 inflammasome-mediated IL-1β, resulting in the formation of larger plaques (Figure 4).

**Table 1:** Evidence supporting the link between gut microbiota and atherosclerosis ^a^.

Bacteria	Source	Methodology	References
*Collinsella* spp., Enterobacteriaceae, Streptococcaceae, *Klebsiella* spp. (*Eubacterium* spp., *Roseburia* spp., Ruminococcaceae)	Human	Metagenome sequencing	[130]
(*Bacteroides xylanisolvens*, *Odoribacter splanchnicus*, *Eubacterium eligens*, *Roseburia inulinivorans*, *Roseburia intestinalis*)	Human	Metagenome sequencing	[131]
Firmicutes phylum (Bacteroidetes)	Human	16S rRNA sequencing	[132]
*Streptococcus* spp., *Escherichia coli*, *Lactobacillus salivarius*, *Solobacterium moorei*, *Atopobium parvulum*, *Ruminococcus gnavus*, *Eggerthella lenta* (*Bacteroides* spp., *Prevotella* spp., *Roseburia intestinalis*, *Faecalibacterium prausnitzii*, *Bacteroides* spp., *Prevotella copri*, *Alistipes shahii*)	Human	Metagenome sequencing	[133]
*Escherichia coli*, *Shigella* spp., *Enterococcus* spp. (*Faecalibacterium* spp., *Subdoligranulum* spp., *Roseburia* spp., *Eubacterium rectale*)	Human	16S rRNA sequencing	[134]
*Clostridium* sp. HGF2, *Streptococcus* sp. M334, *Streptococcus* sp. M143	Human	Metagenome sequencing	[135]
*Faecalibacterium prausnitzii*, *Prevotella copri* (*Bacteroides vulgatus*, *Bacteroides dorei*)	Human	16S rRNA sequencing	[136]
(*Bacteroides xylanisolvens*, *Odoribacter splanchnicus*, *Eubacterium eligens*, *Roseburia inulinivorans*, *Roseburia intestinalis*)	Human	16S rRNA sequencing	[137]
*Enterorhabdus* spp., *Romboutsia* spp., *Proteus* spp., *Eubacterium nodatum*, *Escherichia* spp., *Shigella* spp., *Eubacterium coprostanoligenes*, *Parasutterella* spp., *Muribaculum* spp., *Enterococcus* spp. (*Bifidobacterium* spp., *Alistipes* spp.)	Mice	16S rRNA sequencing	[138]
*Proteobacteria*, *Firmicutes*, *Romboutsia* spp., *Lactococcus* spp. (*Bacteroidetes*, *Actinobacteria*, *Faecalibaculum* spp., *Bifidobacterium* spp., *Bacteroides* spp., *Parabacteroides* spp., *Alloprevotella* spp., *Alistipes* spp., *Odoribacter* spp., *Allobaculum* spp.)	Mice	16S rRNA sequencing	[139]
Firmicutes, *Faecalibaculum* spp., *Oscillibacter* spp., *Eubacterium coprostanoligenes*-group, *Blautia* spp. (*Muribaculaceae*, *Lactobacillus* spp., *Ileibacterium* spp., *Bifidobacterium* spp.)	Mice	16S rRNA sequencing	[140]
Firmicutes, Bacteroidota, Lactobacillaceae, *Lactobacillus* spp., *Helicobacter* spp. (Lachnospiraceae, *Roseburia* spp.)	Mice	16S rRNA sequencing	[141]
Firmicutes, Bacteroidota, Verrucomicrobia, Ruminococcaceae, Bacteroidaceae, *Bacteroides* spp., *Akkermansia* spp. (Rikenellaceae)	Mice	16S rRNA sequencing	[142]
Firmicutes, *Lactobacillus* spp. (Bacteroidetes; *Bifidobacterium* spp.)	Mice	16S rRNA sequencing	[143]

^a^ Names in brackets refer to less dominant microbiota.

## 7. Limitations in Studies Linking Gut Microbiota to Atherosclerosis

Adequate evidence exists linking the gut microbiome to AS. However, the inconsistency in species identifications, mainly due to variations in identification techniques, makes it difficult to ascribe AS and aging to a specific group of gut microbiota. The discrepancies in data are also due to differences in diets and the influence of external factors such as stress and genetic background. Many studies rely on 16S rRNA sequencing to compare species and strain variations. Although useful in some studies, this technique has a poor discriminatory ability for some genera and does not identify variations amongst strains in the same taxon. The inability to identify small genetic variations in strains associated with AS renders it difficult, if not impossible, to understand intercellular interactions and develop a drug that would suppress the growth of atherosclerogenic gut bacteria. Most gut microbiome studies are performed on a small group of individuals, often from the same geographic region, following a specific diet. Findings are seldom followed up over extended periods and do not provide data on changes in the gut microbiome, e.g., the adaptation of gut microbiota to specific treatments. This is important to understand the bidirectional relationship between gut microbiota, aging, and cardiovascular health. Longitudinal follow-up studies will also determine whether changes in the gut microbiome are due to interventions or due to age-related changes, e.g., the development of AS. A typical example is changes observed in TMAO levels and the clarification of the findings. Is an increase in TMAO causing AS, or is it a marker pointing to other underlying medical conditions? Do we have a solid understanding of conditions leading to intestinal inflammation, an aging epithelium, and the translocation of bacteria and their products into the bloodstream that may exacerbate AS? By addressing these and other limitations in study designs, we may have a more comprehensive understanding of the complex interplay between gut microbiota and atherosclerosis, potentially leading to more effective prevention and treatment strategies.

## 8. Future Directions

More research is required on the aging gut wall and metabolites that cause AS. Changes in gut microbiota need to be studied using metagenomics, metabolomics, and proteomics. We need to understand how age-related changes in immune responses (immunosenescence) relate to dysfunctional ECs and the development of AS. Research needs to focus on conducting more targeted interventions aimed at drug design, slowing immunosenescence, and AS. The focus should be on selectively eliminating or modulating “senescent” T and B cells associated with chronic inflammation and vascular damage. We need to identify specific inflammatory markers and signalling pathways involved in these processes. The role of NAD+ (specifically its decline with age) in AS must be studied. Further studies need to be performed on cyclin-dependent kinase inhibitors, senescence-associated β-galactosidase, the proteins p53, p21cip1, ataxia-telangiectasia mutated protein, ataxia telangiectasia, and Rad3-related protein, and phosphorylated histone H2AX as markers of cellular senescence. We need to determine why cells lose their ability to grow when in a senescent state.

## 9. Conclusions

Accumulating evidence from preclinical and clinical studies has shown that the gut microbiome plays an important role in host health and disease. The more we learn about microbial interactions with intestinal epithelial cells and endothelial cells (ECs) in arteries, the closer we become to developing reporter systems to identify atherosclerosis at an earlier stage and develop drugs to treat cardiovascular diseases (CVDs). Microbial-derived biomarkers such as lipopolysaccharides (LPS) and trimethylamine-N-oxide (TMAO) are indicators of atherosclerosis (AS), but other reporters need to be developed. The elderly are more prone to developing AS. The reason for this may be attributed to a changing gut microbiome, associated with a decline in butyrate-producing bacteria. Binding of butyrate to peroxisome proliferator-activated receptor γ (PPARγ) increases the activity of IkBα (nuclear factor kappa-B, NF-κB, inhibitor). This suppresses the production of pro-inflammatory cytokines that cause AS and activates endothelial nitric oxide synthase (eNOS), leading to an increase in nitric oxide (NO), which dilates arteries. Butyrate may regulate mitogen-activated protein kinase (MAPK) to suppress inflammation, muscle cell growth, apoptosis, and the uptake of oxidised low-density lipoprotein (ox-LDL) by macrophages. Butyrate suppresses inflammation by inhibiting interferon γ (IFN-γ), Toll-like receptor 2 (TLR2), and platelet glycoprotein (CD36) signalling. By suppressing CD36, the uptake of ox-LDL by macrophages is prevented, and plaque formation is repressed. Furthermore, butyrate suppresses the NOD-, LRR-, and Nod-like receptor protein 3 (NLRP3) inflammasome, which alleviates inflammation and scar tissue formation. TET methylcytosine dioxygenase 2 (TET-2)-deficient macrophages increase the release of NLRP3 inflammasome-mediated IL-1β, resulting in the formation of larger plaques. The inhibition of interferon gamma (INF-γ) and NLRP3 prevents plaque formation. The elderly are more prone to chronic inflammation, including the recruitment of immune cells to damaged ECs, the deposition of foam cells on the intima, and plaque formation. Furthermore, the release of senescence-associated secretory phenotype (SASP) factors from aged vascular smooth muscle cells (VSMCs) induces the production of pro-inflammatory cytokines and stimulates foam cells to infiltrate arteries. Elevated levels of SASP and matrix metalloproteinase-9 (MMP-9) stimulate the lysis of collagen and the degradation of elastin, potentially causing plaque rupture and thrombosis. The release of damage-associated molecular patterns (DAMPs), free radicals, and reactive oxygen species (ROS) enhances inflammation and deprives the myocardium of O_2_, potentially causing myocardial infarction (MI). As butyrate levels decline, the growth of VSMCs is less controlled, and the uptake of ox-LDL by VSMCs is not prevented. Maintaining butyrate-producing bacteria in the gastrointestinal tract may suppress AS.
